# Supplementary material for: Strain Rate Changes during Stress Echocardiography Are the Most Accurate Predictors of Significant Coronary Artery Disease in Patients with Previously Treated Acute Coronary Syndrome
Source: Diagnostics (Basel). 2023 May 19;13(10):1796. doi: 10.3390/diagnostics13101796 (PMC10217636; doi:10.3390/diagnostics13101796)
Supplement: Supplementary file 1 [file diagnostics-13-01796-s001.zip › diagnostics-2329529-supplementary.pdf]

Supplementary Data

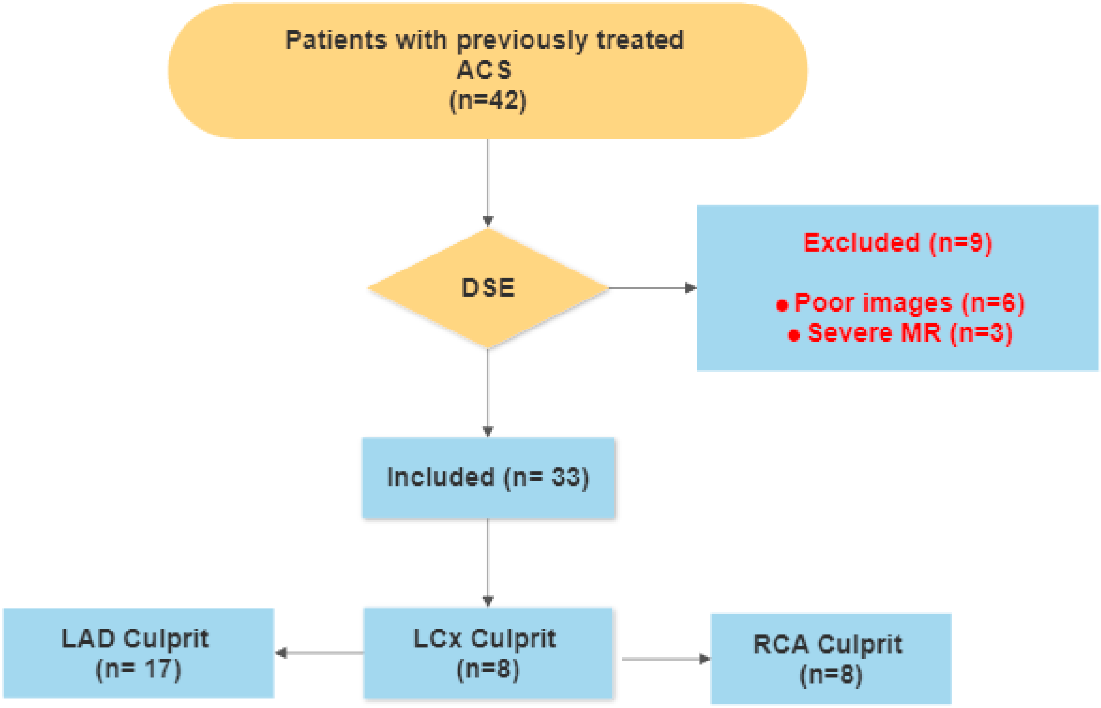

Figure S1. Patients` Flow chart

Table S1. Baseline and stress echocardiographic indices

Variable		Patients with suspected CAD (n=33)
LV systolic function		
LV EF (%)	Rest	36.2 ± 8.9
	Stress	42.2 ± 11
	Delta	-6.03 ± 4.2
	P value	<0.001
Global PSS	Rest	-10.4 ± 2.6
	Stress	-12.2 ± 2.7
	Delta	-1.81 ± 0.9
	P value	<0.001
Global SR	Rest	-0.55 ± 0.2
	Stress	-0.78 ± 0.3
	Delta	-0.23 ± 0.6
	P value	0.01
Territories supplied by LAD		
Longitudinal PSS	Rest	-9.85 ± 2.8
	Stress	-11.1 ± 3.5
	Delta	-1.37 ± 1.5
	P value	<0.001
Strain rate	Rest	-0.56 ± 0.3
	Stress	-0.69 ± 0.4
	Delta	-0.13 ± 0.4
	P value	0.03
Territories supplied by LCx		
Longitudinal PSS	Rest	-10.8 ± 2.9
	Stress	-12.8 ± 3.1
	Delta	-1.83 ± 1.1
	P value	<0.001
Strain rate	Rest	-0.51 ± 0.2
	Stress	-0.79 ± 0.3
	Delta	-0.28 ± 0.2
	P value	<0.001
Territories supplied by RCA		
Longitudinal PSS	Rest	-10.7 ± 3.1
	Stress	-12.5 ± 3.1
	Delta	-1.65 ± 1.2
	P value	<0.001
Strain rate	Rest	-0.54 ± 0.3
	Stress	-0.78 ± 0.3
	Delta	-0.24 ± 0.4
	P value	<0.001

Global WMSI	Rest	2.07 ± 0.5
	Stress	1.85 ± 0.5
	Delta	0.22 ± 0.2
	P value	0.01
WMSI supplied by LAD	Rest	2.24 ± 0.6
	Stress	1.97 ± 0.7
	Delta	0.27 ± 0.3
	P value	0.001
WMSI supplied by LCx	Rest	1.69 ± 0.5
	Stress	1.49 ± 0.4
	Delta	0.20 ±0.4
	P value	0.01
WMSI supplied by RCA	Rest	2.27 ± 0.9
	Stress	2.09 ± 1.0
	Delta	0.11 ± 0.3
	P value	0.04

Table S2. Baseline and stress echocardiographic indices in patients with and without CAD

Variable		Patients LAD - (n=16)	Patients LAD + (n=17)	P value	Patients LCx- (n=25)	Patient LCx+ (n=8)	P value	Patient RCA- (n=25)	Patient RCA+ (n=8)	P value
LV systolic function										
LV EF (%)	Rest	42.3 ± 6.8	30.2 ± 9.6	0.02	38.7 ± 8.5	30.1 ± 8.9	0.03	41.1 ± 9.3	31.5 ± 7.2	0.04
	Stress	51.2 ± 10	33.4 ± 10	<0.001	45.3 ± 11	34.2 ± 10	0.01	49.6 ± 11	37.4 ± 10	0.01
	Delta	8.9± 4.3	3.2 ± 3.5	0.005	6.7 ± 4.3	4.1 ± 3.3	0.03	8.5 ± 4.6	5.9 ± 4.2	0.02
	P	<0.001	0.03		0.003	0.02		<0.001	<0.001	
Global PSS	Rest	-11.4 ± 2.2	-9.10 ± 2.5	0.02	-10.9 ± 2.5	-9.1 ± 2.4	0.04	-11.6 ± 2.6	-10.0 ± 1.8	0.07
	Stress	-14.3 ± 2.6	-10.0 ± 2.7	0.001	-13.0 ± 2.6	-10.0 ± 2.5	0.01	-13.9 ± 2.8	-11.1 ± 2.7	0.01
	Delta	-2.9 ± 0.9	-0.9 ± 1.1	0.02	-2.1 ± 1.2	-0.9 ±1.1	0.04	-2.30 ± 0.9	-1.10 ± 1.2	0.04
	P	0.001	0.04		0.01	0.03		0.001	0.001	
Global SR	Rest	-0.60 ± 0.2	-0.50 ± 0.2	0.04	-0.59 ± 0.2	-0.37 ± 0.1	0.03	-0.68 ± 0.2	-0.51 ± 0.3	0.01
	Stress	-0.89 ± 0.3	-0.67 ± 0.2	0.01	-0.92 ± 0.2	-0.51 ± 0.2	0.001	-0.97 ± 0.2	-0.65 ± 0.3	0.001
	Delta	-0.29 ± 1.1	-0.17± 0.2	0.02	-0.33 ± 0.2	-0.14± 0.2	0.02	-0.29 ± 0.2	-0.14 ± 0.2	0.01
	P	0.01	0.02		0.01	0.03		0.006	0.03	
Territories supplied by LAD										
Longitudinal PSS	Rest	-11.2 ± 2.2	-8.47 ± 2.5	0.002	-9.98 ± 2.7	-9.14 ± 3.5	0.19	-10.6 ± 2.9	9.62 ± 2.6	0.27
	Stress	-13.2 ± 3.1	-9.56 ± 3.1	0.001	-11.6 ± 3.4	-10.0 ± 3.1	0.22	-12.5 ± 3.4	-10.9 ± 3.8	0.11
	Delta	-1.73 ± 0.9	-1.0 ± 0.9	0.01	-1.62 ± 1.4	-0.86 ± 0.9	0.05	-1.9 ± 1.4	-1.17 ± 1.6	0.33
	P	0.001	0.001		0.001	0.01		0.001	0.001	
Strain Rate	Rest	-0.60 ± 0.3	-0.51 ± 0.6	0.02	-0.55 ± 0.3	-0.49 ± 0.2	0.16	-0.58 ± 0.2	-0.61 ± 0.3	0.73
	Stress	-0.77 ± 0.3	-0.52 ± 0.4	0.001	-0.69 ± 0.3	-0.60 ± 0.6	0.40	-0.64 ± 0.4	-0.69 ± 0.4	0.38
	Delta	-0.17± 0.2	-0.02 ± 0.3	0.01	0.14 ± 0.3	-0.11 ± 0.5	0.62	-0.06 ± 0.4	-0.08 ± 0.2	0.54
	P	<0.001	0.06		<0.001	0.001		0.04	0.04	
Territories supplied by LCx										
Longitudinal PSS	Rest	-11.6 ± 2.5	-10.1 ± 3.2	0.08	-11.5 ± 2.8	-8.62± 2.9	0.01	-12.2 ± 3.0	-10.9 ± 2.1	0.12
	Stress	-13.5 ± 2.6	-12.1 ± 3.5	0.04	-13.8 ± 2.5	-9.33± 3.2	0.001	-14.0 ± 3.2	-12.6 ± 2.5	0.09
	Delta	-1.89 ± 1.1	-1.77 ± 1.1	0.11	-2.3 ± 1.1	-0.7 ± 0.3	0.01	-1.80 ± 1.0	-1.70 ± 1.1	0.36
	P	<0.001	0.001		<0.001	0.04		<0.001	0.001	

Strain rate	Rest	-0.54 ± 0.2	-0.48 ± 0.2	0.12	-0.62 ± 0.3	-0.41 ± 0.2	0.01	-0.53 ± 0.3	-0.62 ± 0.2	0.23
	Stress	-0.87 ± 0.3	-0.77 ± 3.4	0.22	-0.95 ± 0.2	-0.51 ± 0.1	0.001	-0.84 ± 0.3	-0.89 ± 0.3	0.52
	Delta	-0.33 ± 0.3	-0.29± 0.3	0.09	-0.31± 0.3	-0.10 ± 0.4	0.01	-0.31 ± 1.6	-0.27 ± 0.9	0.31
	P	0.001	0.001		<0.001	0.01		0.001	0.001	
Territories supplied by RCA										
Longitudinal PSS	Rest	-11.5 ± 3.4	-9.9 ± 2.6	0.03	-12.7 ± 3.2	-11.7 ± 2.7	0.38	-12.1 ± 3.3	-9.71 ± 2.1	0.001
	Stress	-13.5 ± 3.3	-11.1 ± 3.4	0.01	-13.9 ± 3.4	-12.9 ± 3.3	0.14	-14.8 ± 0.3	-10.1 ± 0.3	0.001
	Delta	-2.4 ± 0.9	-1.2 ± 1.1	0.004	-1.21 ± 1.7	-1.20 ± 1.6	0.78	-2.70 ± 1.6	-0.66 ± 0.9	0.01
	P	0.001	0.02		0.04	0.04		0.001	0.01	
Strain Rate	Rest	-0.57 ± 0.3	-0.51 ± 0.3	0.09	-0.72 ± 0.3	-0.71 ± 0.3	0.21	-0.70 ± 0.2	-0.56 ± 0.3	0.03
	Stress	-0.81 ± 0.4	-0.75 ± 0.4	0.31	-0.59 ± 0.3	-0.79 ± 0.3	0.28	-0.98 ± 0.3	-0.61 ± 0.3	0.001
	Delta	-0.24 ± 0.1	-0.24± 0.2	0.44	0.13 ± 0.2	-0.08± 0.2	0.07	-0.26 ± 0.5	-0.05 ± 0.2	0.01
	P	0.001	0.001		0.04	0.08		0.01	0.09	
Wall motion score										
Global WMSI	Rest	1.71 ± 0.4	1.98 ± 0.5	0.17	2.01 ± 0.5	2.28 ± 0.5	0.11	2.04 ± 0.5	2.21 ± 0.4	0.23
	Stress	1.28 ± 0.4	1.83 ± 0.5	0.04	1.74 ± 0.5	2.07 ± 0.3	0.02	1.81 ± 0.5	1.80 ± 0.4	0.44
	Delta	0.43 ± 0.2	-0.16± 0.2	0.13	0.27 ± 0.2	0.21± 0.2	0.36	0.27 ± 0.2	0.40 ± 0.2	0.06
	P	0.01	0.04		0.02	0.02		0.02	0.02	
WMSI supplied by LAD	Rest	1.84 ± 0.66	2.61 ± 0.3	0.001	2.24 ± 0.6	2.25 ± 0.5	0.32	2.26 ± 0.6	2.12 ± 0.7	0.29
	Stress	1.51 ± 0.66	2.39 ± 0.3	0.001	2.00 ± 0.7	1.98 ± 0.7	0.19	1.98 ± 0.6	1.91 ± 0.8	0.49
	Delta	0.32 ± 0.2	0.22 ± 0.3	0.11	0.24 ± 0.3	0.27 ± 0.4	0.11	0.28 ± 0.2	0.21 ± 0.3	0.24
	P	0.01	0.02		0.03	0.04		0.01	0.01	
WMSI supplied by LCx	Rest	1.65 ± 0.46	1.75 ± 0.5	0.12	1.62 ± 0.5	1.95 ± 0.2	0.02	1.28 ± 0.4	1.17 ± 0.4	0.19
	Stress	1.20 ± 0.3	1.32 ± 0.4	0.22	1.29 ± 0.4	1.43 ± 0.4	0.10	1.28 ± 0.4	1.16 ± 0.4	0.21
	Delta	0.44 ± 0.3	0.43 ± 0.4	0.61	0.37 ± 0.3	0.52 ± 0.3	0.04	0.38 ± 0.4	0.66 ± 0.5	0.04
	P	0.003	0.003		0.001	<0.001		0.001	<0.001	
WMSI supplied by RCA	Rest	2.23 ± 0.5	2.31 ± 0.8	0.45	2.16 ± 0.9	2.66 ± 0.7	0.08	2.16 ± 0.9	2.69 ± 0.5	0.03
	Stress	2.17 ± 0.3	2.22 ± 0.7	0.33	2.01 ± 1.0	2.61 ±0.8		2.18 ± 0.9	2.32 ± 1.0	0.11
	Delta	0.06 ± 0.3	0.09 ± 0.2	0.28	0.08 ± 0.3	0.04± 0.2	0.33	0.02 ± 0.2	0.37 ± 0.5	0.02
	P	0.22	0.31		0.03			0.12	0.001	

Abbreviations: EF: Ejection fraction; PSS: Peak systolic strain; LAD: Left anterior descending artery; LCx: Left cirumflex artery; RCA: Right coronary artery; WMSI: wall motion score index;

Table S3. Correlation of significant stenosis with echocardiographic indices

Variable	R	P value
LAD stenosis		
Delta PSS (supplied by LAD)	0.51	<0.001
Delta SR (supplied by LAD)	0.60	<0.001
Delta WMSI (supplied by LAD)	0.39	0.01
Delta global PSS	0.43	0.02
Delta global SR	0.52	0.001
Delta EF	0.50	0.001
Delta global WMSI	0.44	0.02
LCx stenosis		
Delta PSS (supplied by LCx)	0.49	0.001
Delta SR (supplied by LCx)	0.55	<0.001
Delta WMSI (supplied by LCx)	0.35	0.03
Delta global PSS	0.46	0.02
Delta global SR	0.49	0.01
Delta EF	0.51	0.01
Delta global WMSI	0.37	0.04
RCA stenosis		
Delta PSS (supplied by RCA)	0.43	0.01
Delta SR (supplied by RCA)	0.56	<0.001
Delta WMSI (supplied by RCA)	0.26	0.04
Delta global PSS	0.40	0.02
Delta global SR	0.45	0.01
Delta EF	0.47	0.01
Delta global WMSI	0.30	0.03

Abbreviation: EF: Ejection fraction; PSS: Peak systolic strain LAD: Left anterior descending artery; LCx: Left cirumflex artery; RCA: Right coronary artery; WMSI: wall motion score index;

Table S4. Echocardiographic predictors of culprit lesion

**Variable**	**Univariate predictors OR (95% CI)**	**P value**	**Multivariate predictors OR (95% CI)**	**P value**
**In predicting culprit LAD**				
Delta PSS (supplied by LAD)	1.334 (1.161 to 3.315)	0.02	1.134 (1.059 to 3.315)	0.024
Delta SR (supplied by LAD)	1.806 (1.252 to 4.013)	0.001	1.566 (1.191 to 9.013)	0.001
Delta WMSI (supplied by LAD)	1.229 (0.637 to 9.101)	0.31		
**In predicting culprit LCx**				
Delta PSS (supplied by LCx)	1.270 (1.329 to 4.205)	0.01	1.166 (1.029 to 4.205)	0.038
Delta SR (supplied by LCx)	1.808 (1.356 to 6.712)	0.001	1.911 (1.209 to 9.012)	0.001
Delta WMSI (supplied by LCx)	1.120 (1.081 to 9.111)	0.04	1.240 (0.709 to 6.018)	0.391
**In predicting culprit RCA**				
Delta PSS (supplied by RCA)	1.690 (1.227 to 5.003)	0.01	1.450 (1.099 to 6.103)	0.019
Delta SR (supplied by RCA)	2.412 (1.656 to 9.097)	0.01	2.108 (1.613 to 5.133)	0.012
Delta WMSI (supplied by RCA)	1.120 (0.531 to 8.011)	0.29		

Abbreviation: LAD: Left anterior descending artery; LCx: Left cirumflex artery; RCA: Right coronary artery; PSS: Peak systolic strain; SR; Strain rate; WMSI: wall motion score index;
